# Address

**Published:** 1885-08

**Authors:** R. C. Morgan


### Address.

DR. R. C. MORGAN.

*Gentlemen of the Kentucky State Dental Association:*

We should be thankful to the author of our being for His infinite love and mercy in thus permitting us to meet again at an annual convocation--the fifteenth of the Association. I hope I express the feeling of all present, in saying that nothing can be more befitting in convening this evening than to offer Him our just tribute of praise for His unceasing care over us.

We have met in the capacity of an association for mutual benefit. I trust the spirit of brotherly love will abide with us.

In looking back fifteen years, we note that many who once sat with us are missing from our ranks. Some of whom have joined a higher association than is possible for earth.

I hope the prominent object of the meeting is for an earnest, true, and faithful discharge of all the duties that the Association imposes upon us. On this point, we are certainly agreed. But are we a unit as to how this shall be done?

*Promptness* is an indispensable feature in the success of society work. Indeed, in every work in which men are engaged.

Then, when the hour of our meetings is fixed, let us be actuated by the spirit of business—prompt, energetic, active business.

In the preamble of the charter of this body it is stated that "The objects of this Association shall be to cultivate the science and art of Dentistry and all its collateral branches, etc."

Then, that is what we are here for this evening. I raise the question, then, how shall this be done?

Like most questions, it is more readily raised than settled.

Can we accomplish this more effectually than to *increase our membership?*

To do this we must have the hearty co-operation of every member of this body. Only by great earnestness and activity can we succeed.

My observation has been that older dentists seldom join dental associations; though we would welcome them at any time. Then, the increase must come from the class of young men, mostly, and the middle-aged.

Up to the time of the passage of our State dental law, it was no easy thing to find young men qualified to enter dental societies. Because many of them having taken no course of study in a dental college, were not eligible to membership, for the constitution requires that he "shall have been three years in the practice of dentistry, exclusive of his term of pupilage." Now the case is quite otherwise, as no one can presume to practice without proper authority. But even had no law of qualification been passed, with the greatly increasing facilities for dental instruction, it is now possible for every one to obtain a sufficient knowledge of dentistry to entitle him to practice.

It follows, then, that the majority of young men practicing under an act to regulate dentistry in this State, should be eligible to membership in this association.

At least so far as a knowledge of dentistry is concerned, there can be no legitimate reason, therefore, why our membership may not be largely increased.

While I am favoring the admission of young men into the association as active and vigorous members, do not understand me to mean others than those who are *young* in *mind* and *spirit.* All, indeed, who are willing to take upon themselves the burden of society work. Nor would I exclude the most aged. We need both old and young, "Old men for council, and young men for war." Especially do I desire to encourage the young, as the older with long experience are strong, and have learned to be courageous.

Then, as I have before stated, the object of this association is for the increase of scientific knowledge, mutual instruction, and brotherly love. For the completion of this object we must avail ourselves of every means possible. No great work can be done without prosecuting it with perseverance and patience. "Let patience have her perfect work." Nothing so pernicious to the completion of any task, as impatience and idleness. We must call laborers into the vineyard. Sow the good seed. If any have a talent let him use it. If others have five, or ten, let him use them. Don't keep your talent hid in the earth, but use it. As much honor is due to him who has only one talent and uses it, as to him who has ten, and uses them.

